# The Dual Role of Zinc in Spinach Metabolism: Beneficial × Toxic

**DOI:** 10.3390/plants13233363

**Published:** 2024-11-29

**Authors:** Veronika Zemanová, Daniela Pavlíková, Milan Novák, František Hnilička

**Affiliations:** 1 Department of Nutrition Management, Crop Research Institute, Drnovská 507, Ruzyně, 16106 Prague, Czech Republic; 2 Department of Agroenvironmental Chemistry and Plant Nutrition, Faculty of Agrobiology, Food and Natural Resources, Czech University of Life Sciences Prague, Kamýcká 129, 16500 Prague, Czech Republic; 3 Department of Botany and Plant Physiology, Faculty of Agrobiology, Food and Natural Resources, Czech University of Life Sciences Prague, Kamýcká 129, 16500 Prague, Czech Republic

**Keywords:** hormesis, micronutrient, photosynthesis, spinach, stress, toxicity

## Abstract

The effects of zinc (Zn) on the physiology of spinach (*Spinacia oleracea* L.) were investigated in a pot experiment with increasing Zn contents in the horticultural substrate (0, 75, 150, and 300 mg Zn kg^−1^). Interactions among nutrients in the substrate solution affected plant vitality, biomass yield, and nutrient content in plants. The water-soluble Zn fraction increased with the Zn dose, rising from 0.26 mg kg^−1^ in the Control to 0.98 mg kg^−1^ in the Zn300 treatment. The most pronounced effects of elevated Zn content were observed for Ca, Mg, and Mn. In spinach, the dual role of Zn was evident through its impact on yield, particularly regarding aboveground biomass. The positive effects of Zn doses up to 150 mg kg^−1^ were supported by the tolerance index (TI). In contrast, the 300 mg kg^−1^ Zn dose exhibited toxic effects, resulting in a 33.3% decrease in the yield of aboveground biomass and a TI value of 0.7. The effects of Zn on nutrient content in aboveground biomass varied with the dose, and the relationship between Zn and P, Fe, Mn, Ca, and K content confirmed a correlation. The toxic effect of the Zn300 treatment was evidenced by a decrease in Ca, Cu, and Fe contents. Additionally, the results of the Zn300 treatment indicated a negative effect on the synthesis of photosynthetic pigments and photosynthesis, likely due to induced oxidative stress. The production of oxalic acid also suggested a toxic effect of the highest Zn dose on spinach.

## 1. Introduction

Zinc (Zn) is an essential trace element for plants, crucial for numerous cellular functions, including metabolic and physiological processes, enzyme activation, and ion homeostasis [1]. Maintaining an optimal Zn content, typically ranging from 30 to 200 mg Zn kg^−1^ dry weight, is essential for ensuring the proper metabolic functioning of most plants [2]. The metabolic functions of Zn are due to its ability to form tetrahedral complexes with N-, O-, and S-ligands, through which it fulfils both catalytic and structural roles in enzymatic reactions [3]. Zinc is an essential part of more than 300 enzymes [4] and is involved in various aspects of enzyme functionality, including serving as a structural component, facilitating protein subunit interactions, participating in catalytic centres, and occupying co-catalytic sites. Zinc acts as a cofactor for numerous enzymes, such as carbonic anhydrase, alkaline phosphatases, aldolases, carboxypeptidases, alcohol dehydrogenases, and superoxide dismutase [5]. Its involvement in cell division, DNA and RNA metabolism, and protein synthesis are well-documented [6]. Additionally, Zn is essential in N metabolism, photosynthesis, and auxin synthesis, particularly in the production of tryptophan, a key precursor of auxin. Furthermore, the interaction between Zn phospholipids and sulfhydryl clusters of membrane proteins contributes to enhancing the stability of the membrane [1].

In a solution culture experiment, the inhibitory effect of macronutrients, including Ca, Mg, K and Na, on Zn absorption by plant roots was confirmed. In soil, their effects appear to be mediated by their influence on soil pH [7]. In plants, Zn activity interacts with several other essential elements, such as Cu, Fe, Mn, Mg, and P, in ways that can be either synergistic or antagonistic, both chemically and biologically. A high P supply in the soil can decrease the availability of Zn for plants by reducing Zn solubility and inhibiting root growth [2]. The distribution and translocation of P from root to shoot are diminished under conditions of high Zn and low P. Phytic acid, the storage form of P in plants, interferes with P and Zn homeostasis. Reducing phytic acid levels leads to an increase in the concentrations of both elements [8].

The Zn content in plants can be influenced by low Zn total soil content (mainly in sandy soils), low organic matter, a high P status, and salt concentrations (saline soils). Zinc deficiency is mainly associated with soil pH; alkaline and/or calcareous soils typically exhibit low available Zn content [7]. The critical threshold for Zn content in plant tissues is less than 20 mg kg^−1^ [9]. Aboveground biomass is generally more adversely affected than root growth. Visible symptoms of Zn deficiency include a reduction in leaf size, internode shortening, interveinal chlorosis, and chlorosis, particularly in young leaves [2]. Zinc limitation results in a deficiency of Zn-dependent enzymes, such as Cu/Zn-superoxide dismutase (SOD) and carbonic anhydrases, leading to decreased quenching of superoxide and increased oxidative stress. Furthermore, Zn deficiency impairs net photosynthesis in plants by disrupting the activity of carbonic anhydrase, the key enzyme limiting CO_2_ fixation in C4 plants [10].

At a very high content of Zn in the soil, Zn toxicity can significantly inhibit plant growth and reduce chlorophyll content in young leaves, resulting in leaf reddening due to increased anthocyanin production [9]. As with most other metals, Zn toxicity is primarily caused by the replacement of other weakly bound metal ions at active sites. These sites may include Mg in chlorophyll, leading to the formation of [Zn]–chlorophyll, which is considerably less effective for plant photosynthesis [2]. Elevated Zn contents also lead to oxidative damage by increasing the accumulation of reactive oxygen species (ROS), such as hydrogen peroxide, superoxide, hydroxyl radicals, and singlet oxygen, which subsequently causes membrane lipid peroxidation [1,11]. To preserve ROS homeostasis, plants employ ROS-scavenging enzymes, the activity of which varies according to Zn levels and the plant organ [12].

Spinach (*Spinacia oleracea* L.) is a leafy vegetable with a higher accumulation of metals, including Zn, and relatively high growth rates. Its changes in metabolism during growth on the contaminated matrix are widely studied [13,14,15,16,17,18]. Therefore, the present study aimed to determine the uptake and accumulation of increasing Zn rates in spinach. The hormetic response induced by Zn was investigated to identify the optimal Zn rate for spinach cultivation. Additionally, the levels of various mineral nutrients in the edible parts of the plant were examined to evaluate any synergistic or antagonistic effects of Zn accumulation in plant tissue on the uptake of these elements.

## 2. Results and Discussion

### 2.1. The Impact of Zn on the Water-Soluble Fraction of Nutrients

**Figure 1:**
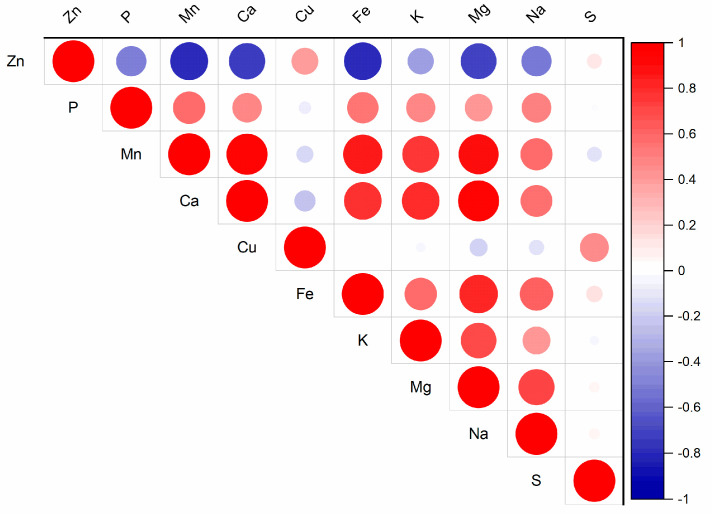
Correlation matrix for the water-soluble fraction of Zn and other nutrients in the substrate (for *p*-values, see Table S2).

Interactions among nutrients in the soil solution can significantly impact plant vitality, biomass yield, and the nutrient content and ratios within plants [19]. These nutrient interactions influence the supply of Zn from soil to plants [20]. In the substrate solution, the relationships among the water-soluble fractions of nutrients were observed (Figure S1). The increasing Zn content in the substrate solution affects the contents of other nutrients (Figure 1, Tables S1 and S2). The water-soluble fraction of Zn increased from 0.26 mg kg^−1^ (Control) to 0.98 mg kg^−1^ (Zn300). The treatment with the highest Zn content contained 3.8 times more Zn in the substrate solution than without any added Zn. Zinc is known to interact with microelements such as Cu, Fe, and Mn [7]. The results from the substrate solution indicated that elevated Zn contents had the most significant effect on Fe and Mn, as well as Ca and Mg (Figure 1). The contents of Fe, Mn, Ca, and Mg in the Zn300 treatment decreased in 47.1, 35.7, 39.3, and 40.9% of the Control content, respectively. Correlation analysis confirmed the relationships between the water-soluble fraction of Zn and the water-soluble fractions of Fe (r = −0.81, *p* < 0.001), Mn (r = −0.81, *p* < 0.001), Ca (r = −0.75, *p* < 0.001), and Mg (r = −0.73, *p* < 0.001). The high Zn content caused a nutritional imbalance. Its presence affects the transport of other elements from the soil solution to plants because the transporter of divalent metal cations, such as Cu, Fe, Mn, and Zn, generally exhibits broad substrate specificity [21]. Zinc diffuses from the soil solution into the empty area within the root cell wall. It is transported through root cells by the Zn-regulated transporter-like proteins family (ZIP) [22,23]. Under high Zn conditions, the activities of the ZIP family transporters, which facilitate Fe uptake and transport, are repressed, leading to Fe deficiency in plants [24]. The antagonistic interactions between Zn and Cu were described by Wolf et al. [25] and Adamczyk-Szabela and Wolf [26]. According to these authors, Cu bioaccumulation is inhibited by the presence of Zn, and the results indicate the involvement of similar carrier sites in the absorption and transport mechanisms of both elements. Despite the observed trend in Cu content with increasing Zn doses in the substrate of our experiment, the Zn-Cu interaction was not confirmed (Tables S1 and S2). Similar results were noted for the Zn-S interaction.

In the substrate solution, the contents of P, K, and Na varied depending on the Zn dose in the substrate (Table S1). However, compared to the Control, a decrease in nutrient levels was observed with the Zn300 treatment, resulting in 86.6, 73.2, and 73.2% of the Control content for P, K, and Na, respectively. Correlation analysis revealed a significant relationship between the water-soluble fraction of Zn and the water-soluble fractions of P (r = −0.52, *p* < 0.05) and Na (r = −0.52, *p* < 0.05). The correlation for K was not significant. These results are partly consistent with previous research indicating that high Zn contents in the soil solution can reduce the bioavailability of P around the root surface, thereby triggering a P starvation signal in plants [27]. According to Boudali et al. [28], high Zn content can inhibit P translocation by upregulating and downregulating the P transporters OsPT2 and OsPT8 in the roots.

Elevated sulphur content in the soil solution was observed in the Zn75 and Zn150 treatments in this experiment. Zinc forms soluble complexes with sulphate ions, affecting the total Zn concentration in the solution [7]. The formation of these complexes may provide protective functions under Zn stress [29].

### 2.2. The Accumulation of Zn in Spinach Biomass and Its Effect on Yield

**Figure 2:**
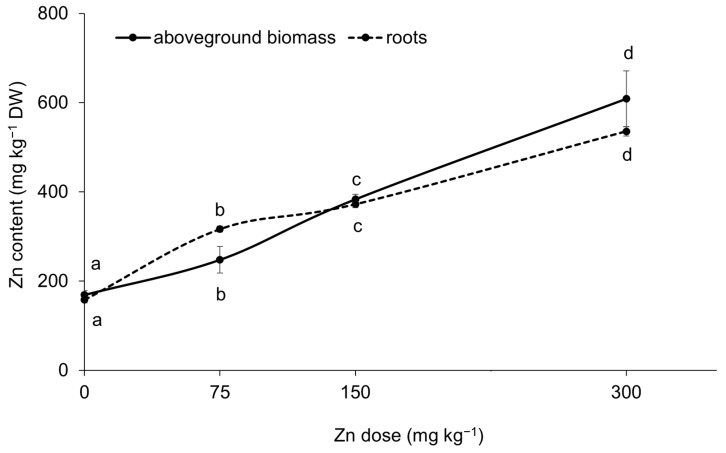
Accumulation of Zn by spinach under increasing Zn doses in the substrate. Data are presented as the mean ± standard deviation (SD) from four biological replicates. Different letters indicate statistically significant differences (*p* < 0.05) among the treatments of spinach aboveground biomass and roots based on Fisher’s LSD test.

**Figure 3:**
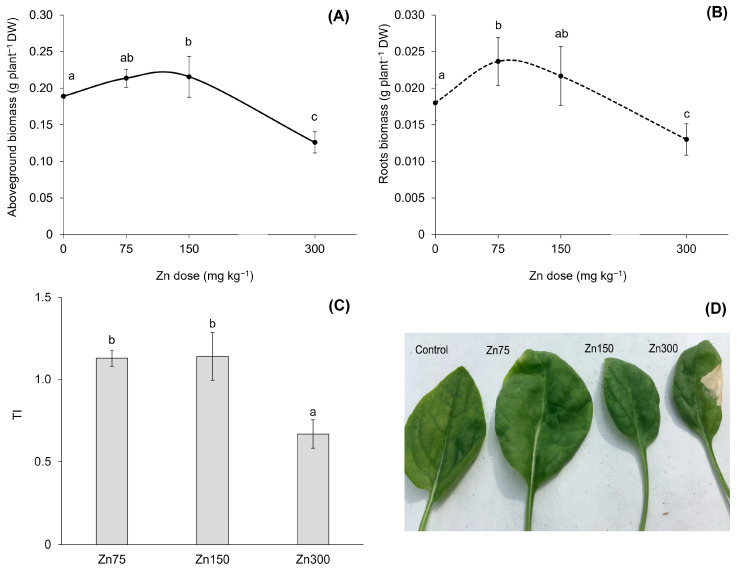
Dry biomass of spinach aboveground biomass affected by increasing Zn doses in the substrate (**A**). Data are presented as the mean ± standard deviation (SD) from four biological replicates. Different lowercase letters indicate statistically significant differences (*p* < 0.05) among the treatments of spinach aboveground biomass and roots based on Fisher’s LSD test. Dry biomass of spinach roots affected by increasing Zn doses in the substrate (**B**). Data are presented as the mean ± standard deviation (SD) from four biological replicates. Different lowercase letters indicate statistically significant differences (*p* < 0.05) among the treatments of spinach aboveground biomass and roots based on Fisher’s LSD test. Tolerance index (TI, expressed as the ratio of individual Zn treatment dry biomass to Control dry biomass) of spinach aboveground biomass (**C**). Data are presented as the mean ± standard deviation (SD) from four biological replicates. Different lower-case letters indicate statistically significant differences (*p* < 0.05) among the treatments of spinach aboveground biomass and roots based on Fisher’s LSD test. Aboveground biomass of spinach (**D**).

Elevated Zn contents in the substrate resulted in increased Zn contents in both the aboveground biomass and roots of spinach (Figure 2). This finding confirmed a strong correlation between the water-soluble fraction of Zn and its content in the aboveground biomass and roots, with correlation coefficients of r = 0.94 and r = 0.93 (*p* < 0.001), respectively. The results indicated a dual role of Zn in spinach physiology and biochemistry, exhibiting both beneficial and toxic effects. In the Zn300 treatment, the Zn content in the aboveground biomass was 3.6 times greater than that of the Control. This elevated content was toxic to the plant, as shown by a 33.3% reduction in dry biomass yield and a tolerance index (TI) value of 0.7 (Figure 3A,C). Additionally, the plants exhibited visual symptoms of Zn-induced toxicity, such as chlorosis and necrosis in young leaves after exposure to a substrate containing 300 mg kg^−1^ of Zn (Figure 3D). For spinach, the significant impact of Zn on dry biomass yield, particularly in the aboveground biomass, was confirmed by the correlation between the Zn content and dry biomass (r = −0.68, *p* < 0.01). Zinc doses up to 150 mg kg^−1^ in the substrate demonstrated hormetic effects, as indicated by the growth response of spinach (Figure 3A,B). The positive effects of the Zn75 and Zn150 treatments were further supported by the TI results (Figure 3C). The hormetic effect is a well-documented dose-response phenomenon that can arise from exposure to essential elements [30,31]. At low doses, these elements can promote the growth of organisms, while at high doses, they can inhibit various biological activities. Leafy vegetable crops, particularly spinach, are sensitive to elevated Zn contents due to their inherent capacity for high Zn uptake [32]. Alia et al. [13] observed a significant reduction in the biomass of spinach at Zn concentrations of 250, 500, and 750 mg kg^−1^. Specifically, exposure to higher doses of Zn decreased aboveground biomass and root weights (both fresh and dry) of 23%, 44%, 6%, and 14%, respectively, compared to the Control groups. Barman et al. [33] demonstrated that high Zn concentrations, particularly those exceeding 500 mg Zn kg^−1^, could adversely affect plant yields by inducing nutrient deficiencies. Plant growth inhibition under stress conditions is attributed to disturbed nutrient uptake, low water potential, and oxidative stress [14].

Oxidative stress can be induced by the generation of ROS resulting from the high supply of Zn in plants. In contrast, at optimal levels, Zn acts as an antioxidant [12]. To maintain ROS homeostasis, plants employ ROS-scavenging enzymes, such as catalases (CATs), SODs, ascorbate peroxidases (APX), glutathione peroxidases (GPX), and peroxiredoxins. The activities of these enzymes vary to different extents depending on Zn concentrations and the specific plant organ. Under conditions of Zn toxicity (500, 1000, and 1500 µM), the activities of SOD, CAT, GPX, and glutathione reductase were either increased or not significantly affected, while at 2000 µM Zn, the activity of these enzymes was adversely impacted. The enhanced activity of total SODs and total APX in plant cells under moderate Zn toxicity clearly indicates an increased production and elimination rate of superoxide radicals and hydrogen peroxide, respectively. However, the highest Zn concentration significantly decreased enzyme activity [34]. Additionally, elevated Zn contents suppressed leaf and root elongation, indicating that high Zn contents may interfere with the normal division of cells [11]. The results of the Zn300 treatment in the spinach experiment confirmed this finding.

### 2.3. The Effect of Zn on Nutrient Composition in Spinach Aboveground Biomass

**Figure 4:**
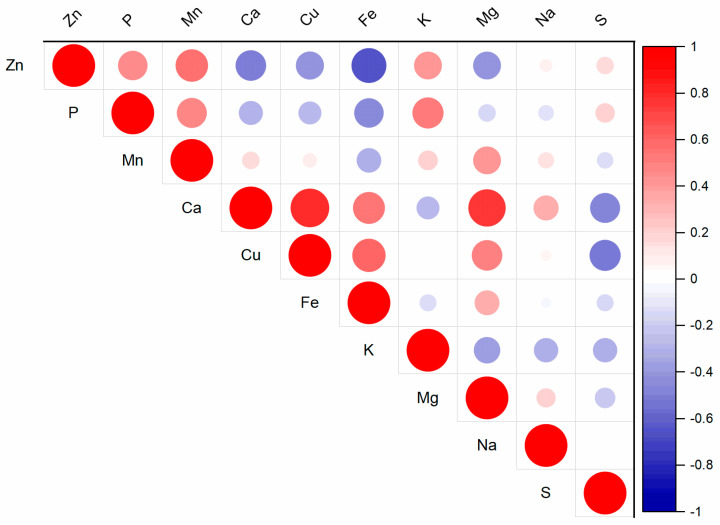
Correlation matrix for Zn content and other nutrients in the aboveground biomass of spinach (for *p*-values, see Table S3).

The results of this study indicate that higher Zn contents lead to changes in the nutrient composition of spinach aboveground biomass (Table 1). The elements Cu, Fe, Mg, and Zn are essential for plant growth and metabolic processes [5]. These elements interact within the rhizosphere and plant metabolism, and their interactions influence their uptake from the soil [35]. An excess of Zn has been shown to decrease the uptake of these elements in plants [36]. The effect of varying Zn doses on Ca, Fe, and Mn content confirmed a correlation between these elements and Zn content in spinach aboveground biomass (Figure 4, Table S3), with correlation coefficients of r = −0.51 (*p* < 0.05), −0.66 (*p* < 0.01), and 0.57 (*p* < 0.05), respectively. A clear trend was observed with increasing Zn doses: Fe decreased by 12.9–32.7%, while P increased by 5.8–17% in the aboveground biomass; however, not all changes were statistically significant (Table 1). The changes in other nutrients within the aboveground biomass varied according to the Zn dose. Notably, the Zn300 treatment exhibited a significant impact compared to the Control, affecting the content of Mn, Ca, Cu, and K. Specifically, Mn increased by 31%. In comparison, Ca decreased by 33.8%, Cu decreased by 19.4%, and K increased by 7.7%. The negative effects of Zn doses were also reflected in the Mg content, which declined by 7.7% to 17.7%, although this change was not statistically significant. Zinc can compete with other polyvalent cations to form coordination complexes, potentially inducing mineral nutrient deficiencies [37]. This phenomenon explains the reduced Mg, Mn, and Ca levels observed in Zn-treated plants. The results from this spinach study are consistent with previous findings. According to Barman et al. [33], elevated Zn contents may lead to deficiencies in other nutrients, such as Fe or Mg, due to the similar ion radii of Zn^2+^ and Fe^2+^, as well as Zn^2+^ and Mg^2+^. Fe deficiency can induce chlorosis through reductions in chlorophyll synthesis, chloroplast degradation, and interference with the uptake of Mg, Mn, and P [32]. Chlorosis and chlorophyll degradation were observed in the Zn300 treatment during this spinach experiment, which is associated with a decrease in Fe contents in the aboveground biomass of spinach (Table 1).

According to Alloway [7], several nutrients, such as Ca, K, Mg, and Na, are known to inhibit Zn absorption by plant roots in solution culture experiments. However, their main effect on soils appears to be through their influence on soil pH. This phenomenon explains the ambiguous changes in K and Na content in the aboveground biomass of spinach observed in this experiment. The activity of sulphate uptake in plants is related to the S content in the soil solution. Exposure to elevated metal levels often stimulates the activity of enzymes involved in S metabolism. Increased S content in the soil solution has been found to enhance plant survival under stress by maintaining normal metabolic functions [38]. Sulphur mitigates stress in plants by enhancing tolerance mechanisms by increasing glutathione, non-protein thiols, and phytochelatins [39]. Our results confirm the significance of S for plants. The highest biomass yield was observed in the Zn75 and Zn150 treatments, which contained the highest S contents in both the soil solution and plant biomass.

### 2.4. The Effect of Zn on Chlorophyll Fluorescence and Photosynthesis

**Figure 5:**
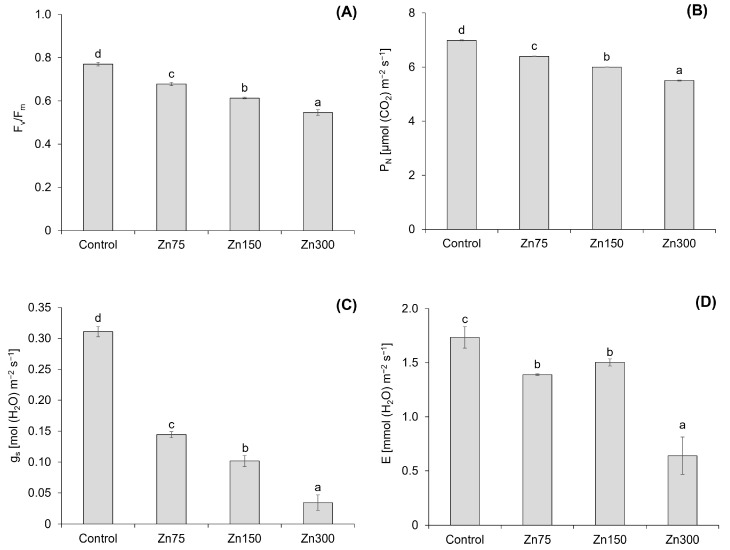
Chlorophyll fluorescence (F_v_/F_m_) in the aboveground biomass of spinach (**A**). Data are presented as the mean ± standard deviation (SD) from four biological replicates. Different letters indicate statistically significant differences (*p* < 0.05) among the treatments of spinach aboveground biomass based on Fisher’s LSD test. Net photosynthetic rate (P_N_), transpiration rate (E), and stomatal conductance (g_s_) in the aboveground biomass of spinach (**B**–**D**). Data are presented as the mean ± standard deviation (SD) from four biological replicates. Different letters indicate statistically significant differences (*p* < 0.05) among the treatments of spinach aboveground biomass based on Fisher’s LSD test.

The results indicate that an increase in Zn content leads to significant changes in the chlorophyll fluorescence (F_v_/F_m_) and photosynthetic parameters—net photosynthetic rate (P_N_), transpiration rate (E), and stomatal conductance (g_s_)—of spinach aboveground biomass (Figure 5). The F_v_/F_m_ decreased by 12.0–29.1% with an increasing Zn dose (Figure 5A), and a strong correlation was confirmed between the Zn content in spinach aboveground biomass and F_v_/F_m_ (r = −0.94, *p* < 0.001). Xu et al. [40] reported a significant decrease in F_v_/F_m_ (*p* < 0.01) with an increasing Zn concentration in *Hydrilla verticillata*. The maximum reduction was observed at the highest Zn level, indicating severe damage to the PS II reaction centre due to Zn toxicity. On the other hand, Repkina et al. [41] reported varying effects of Zn doses on 3 weeks old mustard plants. A low Zn dose (50 mg kg^−1^) increased the F_v_/F_m_, while a medium Zn dose (100 mg kg^−1^) did not affect F_v_/F_m_. In contrast, a high Zn dose (150 mg kg^−1^) decreased the F_v_/F_m_, which is consistent with the results observed in spinach.

The effects of increasing Zn doses on P_N_, E, and g_s_ demonstrated a significant correlation between these parameters and Zn content in the aboveground biomass of spinach. The correlation coefficients were r = −0.95 (*p* < 0.001) for P_N_, r = −0.90 (*p* < 0.001) for E, and r = −0.87 (*p* < 0.001) for g_s_. All three photosynthetic parameters declined with increasing Zn doses (Figure 5B–D), with P_N_ and g_s_ decreasing by 8.4–21.2% and 53.6–89.0%, respectively, while E decreased by 13.3–63.1%. However, not all changes in E were statistically significant (Figure 5D). The reduction in photosynthesis, which results in decreased stomatal conductance and mesophyll conductance of CO_2_, was confirmed by Nath et al. [23]. Repkina et al. [41] also confirmed a decrease in all three studied photosynthetic parameters under Zn doses of 100 and 150 mg kg^−1^ in mustard plants. Zinc toxicity has been shown to reduce both mesophyll and stomatal conductance, resulting in a deficiency of CO_2_ at Rubisco [42]. High Zn contents can substitute for Mn in thylakoid membranes [43], disrupting the efficiency of PSII. Mn-deficient plants are also characterised by tissue necrosis due to decreased MnSOD levels and increased oxygen free radicals [2].

The relationship between the photosynthesis parameter and the growth of spinach, as well as the negative effects of Zn, was confirmed by a correlation between P_N_ and E with the DW of spinach aboveground biomass (r = 0.51, *p* < 0.05 and r = 0.77, *p* < 0.001, respectively). According to Kaur and Garg [4], plants exposed to elevated Zn levels exhibit various photosynthetic responses that depend on species, Zn concentration, and genetic variation. These authors reported that the evidence indicates no single specific target of Zn toxicity in photosynthesis; instead, increased Zn levels may trigger a cascade of events.

### 2.5. The Effect of Zn on Oxalic Acid

**Figure 6:**
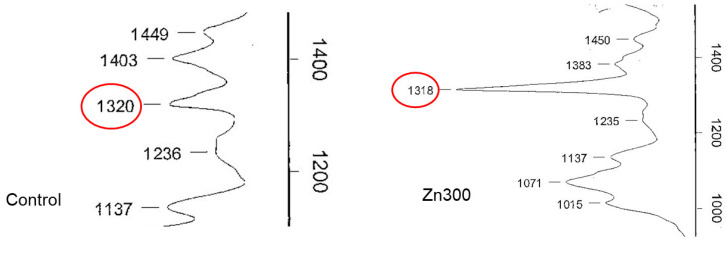
Infrared spectra of oxalic acid in the aboveground biomass of spinach from the Control and Zn300 treatments. The red circle indicates the presence of oxalic acid.

In plants, Zn deficiency and toxicity stimulate the production of Zn chelators, such as oxalic acid. Spinach contains high levels of oxalic acid, ranging from 329.6 to 2350 mg of total oxalates per 100 g of fresh weight [44]. Oxalic acid plays a significant role in various physiological functions, including calcium homeostasis, pH regulation, plant growth, photosynthesis, and the detoxification of toxic elements [44]. It can sequester Zn ions in the cytosol or subcellular compartments, accumulating excess Zn in vacuoles and the cell wall. Subcellular compartmentalisation is an important mechanism for enhancing Zn efficiency [45]. In our experiment, the salts of oxalic acid in spinach were characterised using infrared spectroscopy. The spinach treatments exposed to increased levels of Zn showed a dominant infrared spectral band at 1317–1320 cm^−1^, with the Zn300 treatment showing a more intense band than the Control (Figure 6). These results confirm the role of Zn precipitation by oxalic acid in plant detoxification.

### 2.6. The Impact of Zn in Spinach Aboveground Biomass on Human Health

According to Natasha et al. [3], Zn is essential for living organisms; however, it can become lethal at high levels when the target hazard quotient (THQ) reaches values of 1.5. Consequently, the potential health hazard associated with Zn levels in the edible part of spinach was assessed for both adults and children. The results of the THQ assessment, which evaluates the non-carcinogenic risk to humans across all treatments, are presented in Table 2. THQ values for spinach lower than 1 indicate no significant health risk to either adults or children. Similarly, Barman et al. [33] noted that elevated concentrations of Zn in plants are unlikely to pose a toxicity hazard to the humans or livestock that consume them. The low THQ values observed in this spinach experiment are attributed to the low consumption of spinach in the Czech Republic [46].

## 3. Materials and Methods

### 3.1. Pot Experiment

The pot experiment was performed during April and May in a greenhouse under semi-controlled conditions with 14 ± 1 h day (irradiance of 199 ± 64 W m^−2^) and 10 ± 1 h night photoperiod cycle, day-time temperatures ranging from 20 to 23 °C and night-time temperatures between 15 and 18 °C, and relative humidity of ~60%. A randomised design with four replications for each treatment was used. The experimental plant, spinach (*Spinacia oleracea* L.), was cultivated in a horticultural substrate (AGRO CS: pH 6, average nutrient content: N 200 mg kg^−1^, P 62 mg kg^−1^, K 200 mg kg^−1^; composed of 80% white peat and 20% black peat, with a density of 20 kg substrate m^−3^ and a texture of 0–10 mm; and water-soluble Zn content 0.3 mg kg^−1^). Pots were filled with 1 kg of the substrate, which was mixed with Zn dose according to the treatment: (i) Control—no added Zn, (ii) Zn75—75 mg Zn kg^−1^ substrate, (iii) Zn150—150 mg Zn kg^−1^ substrate, and (iv) Zn300—300 mg Zn kg^−1^ substrate. Zinc was applied as a solution of ZnSO_4_. Spinach seeds were sown in the substrate pots and regularly irrigated. After 8 weeks of growth, the spinach was harvested and separated into aboveground biomass and roots. The biomass was rinsed with distilled water, blotted dry using filter paper, and weighed. Subsequently, it was oven-dried at 40 °C until a constant weight was achieved to determine the dry biomass and then homogenised for nutrient analysis.

### 3.2. Nutrients Determination

The nutrient contents in the water-soluble fraction of the substrate and plant biomass were measured using an Agilent 720 inductively coupled plasma optical emission spectrometer (ICP-OES; Agilent Technologies Inc., Santa Clara, CA, USA) [46] after a respected preparation process. The water-soluble fraction of the nutrients was extracted from the substrate using demineralised water (1:5, *w*/*v*; 30 min shaking; 12 h equilibration; and centrifugation at 5000 rpm). For plant biomass, the homogenised dry biomass (0.5 ± 0.05 g) was mineralised in 10 mL of a HNO_3_ and H_2_O_2_ mixture (4:1, *v*/*v*) using low-pressure microwave digestion with an Ethos 1 device (MLS GmbH, Leutkirch im Allgäu, Germany). Quality assurance was verified by mineralising certified reference material (CRM NIST 1570a Spinach leaves, Analytika^®®^, Prague, Czech Republic) under the same conditions.

### 3.3. Chlorophyll Fluorescence and Photosynthetic Parameters Determination

For chlorophyll fluorescence, the maximum quantum yield of PSII (F_v_/F_m_) was calculated using the formula F_v_/F_m_ = (F_m_ − F_0_)/F_m_, with F_m_ and F_0_ measured by a portable fluorometer (OS1-FL; Opti-Sciences, ADC, BioScientific, Ltd., Hoddesdon, UK) [47]. A fresh leaf was dark-adapted for 20 min using shading clips, then irradiated with a 660 nm solid-state light source, with filters blocking radiation longer than 690 nm. Saturation of the photosystem was achieved with a filtered 35 W halogen lamp (350–690 nm) providing a 15,000 μmol m^−2^ s^−1^ pulse for 0.8 s.

The net photosynthetic rate (P_N_), transpiration rate (E), and stomatal conductance (g_s_) were measured using a portable gas exchange system LCpro+ (ADC BioScientific, Ltd., Hoddesdon, UK) between 8:00 and 13:00 h [47]. Each measurement lasted 10 min after reaching steady-state conditions inside the measurement chamber. The chamber conditions were maintained at 25 °C, an ambient CO_2_ concentration of 550 ± 50 μl L^−1^, an air flow rate of 205 ± 30 μmol s^−1^, and irradiance of 650 ± 50 μmol (photon) m^−2^ s^−1^ for photosynthetically active radiation.

### 3.4. Analyses of Infrared Spectra of Oxalic Acid

The dry biomass of spinach (3–4 g) was extracted using a solution of methanol and ultrapure H_2_O (1:1, *v*/*v*, 10 × 50 mL) and then evaporated to dryness at 40 °C using a rotary evaporator equipped with a water jet pump (Büchi Labortechnic, Flawil, Switzerland). The isolated fraction was characterised using infrared (IR) spectroscopy with an IR spectrometer (Bruker IFS 88, Ettlingen, Germany). The sample fractions were pressed into KBr pellets (approximately 1 mg) under a pressure of 6.86 MPa, with evacuation performed by an oil rotary vacuum pump (Büchi, Labortechnic, Flawil, Switzerland), and measured in cells with a length of 10 mm [48].

### 3.5. Factors Calculation

The tolerant index (TI) was calculated to determine the decrease in plant yield resulting from Zn treatments using the following equation [49]:

TI = DB_Zn_/DB_Control_, (1)

where DB_Zn_ is the dry biomass of the Zn75, Zn150, and Zn300 treatments, and DB_Control_ is the dry biomass of Control. In general, TI values near zero indicate plant sensitivity to contamination, whereas a TI value of one indicates that the plants are unaffected by and tolerate the contamination.

The target hazard quotient (THQ) for Zn was calculated according to Pavlíková et al. [46] for assessing non-carcinogenic human health risks. In the initial step, the THQ for both adults and children was estimated based on chronic daily intake (CDI, mg kg^−1^ day^−1^) using the following equation:

CDI = (C × IR ×EF × ED)/(ET × BW),(2)

where C represents the Zn content in the edible part (mg kg^−1^ dry weight), IR represents the daily consumption of spinach (0.003 kg person^−1^ day^−1^) as reported by the Ministry of Agriculture of the Czech Republic [50], EF represents the exposure frequency (365 and 250 days year^−1^), ED represents the exposure duration (30 and 7 years), ET represents the average time (non-carcinogenic elements: 365 × ED), and BW represents the average body weight (70 and 26 kg).

In the second step, the THQ of Zn, which reflects non-carcinogenic effects, was estimated for consumption and calculated using the following equation:

THQ = CDI/RfD,(3)

where CDI represents the chronic daily intake by consumption (mg kg^−1^ day^−1^), and RfD represents the oral reference consumption dose of Zn (mg kg^−1^ day^−1^). The RfD value of Zn is 0.3 [51]. A THQ less than 1 indicates a negligible risk of exposure, whereas a THQ value greater than 1 indicates the adverse existence of non-carcinogenic health risks associated with exposure [52].

### 3.6. Statistical Analyses

Results were statistically analysed using Statistica 14.0 (Tibco Software, Palo Alto, CA, USA), CANOCO 5.1 (Microcomputer Power, Ithaca, NY, USA), and Origin 2024b (OriginLab Corporation, Northampton, MA, USA). Data from the technical replicates were averaged across four independent biological replicates (pots) per treatment, and results were presented as mean values with standard deviation (SD). The data were tested for homogeneity of variance and normality using Levene’s and Shapiro–Wilk tests. To identify statistically significant differences among treatments, a one-way analysis of variance (ANOVA) followed by Fisher’s LSD test (*p* ≤ 0.05) was applied. The relationships between nutrient contents in the substrate solution and spinach biomass were visualised using a principal component analysis (PCA) diagram and a colour correlation matrix. In the diagram, the vectors’ length and direction indicate the vector effect’s strength and the correlation between the vectors. A long vector for a particular variable substantially impacts the analysis results, while a short vector indicates minimal influence. An angle of <90° between the vectors suggests a positive correlation, while an angle of >90° indicates a negative correlation.

## 4. Conclusions

In this experiment, the dual role of Zn—both beneficial and toxic—in the metabolism of spinach was confirmed. Zinc doses of up to 150 mg kg^−1^ in the substrate exhibited hormetic effects, as indicated by the growth response of the spinach plants. Exposure to the highest dose of Zn resulted in a significant inhibition of biomass growth compared to the Control, indicating a toxic effect of Zn. An increase in Zn content leads to alterations in the nutrient composition of the aboveground biomass of spinach. The relationship between Zn and Ca, Fe, and Mn content confirmed a correlation in the aboveground biomass. Additionally, the highest dose of Zn may result in deficiencies of other nutrients, such as Mg or Fe, due to the similar ion radius of Zn^2+^ and Fe^2+^, as well as Zn^2+^ and Mg^2+^. Chlorosis, characterised by chlorophyll synthesis and chloroplast degradation reductions, was observed in the Zn300 treatment. These changes, along with alterations in photosynthesis, indicate a toxic effect of high Zn treatment and suggest that Zn content in biomass greater than 400 mg kg^−1^ is toxic for spinach. The toxicity of the highest Zn dose also suggests the production of oxalic acid.

## Figures and Tables

**Table 1:** Content of nutrients (mg kg^−1^ DW) in the aboveground biomass of spinach. Data are presented as the mean ± standard deviation (SD) from four biological replicates. Different lowercase letters indicate statistically significant differences (*p* < 0.05) among the treatments of spinach aboveground biomass and roots based on Fisher’s LSD test.

Nutrient (mg kg^−1^ DW)	Control	Zn75	Zn150	Zn300
Ca	11,724.6 ± 1703.2 ^b^	7968.0 ± 2047.9 ^a^	7649.2 ± 479.5 ^a^	7766.0 ± 1305.7 ^a^
Cu	4.0 ± 0.3 ^b^	3.2 ± 0.4 ^a^	2.9 ± 0.4 ^a^	3.3 ± 0.2 ^a^
Fe	87.2 ± 23.4 ^b^	76.0 ± 7.3 ^ab^	69.6 ± 3.4 ^ab^	64.0 ± 10.6 ^a^
K	88,218.5 ± 4868.0 ^a^	91,327.2 ± 4946.6 ^ab^	89,264.3 ± 3084.2 ^ab^	95,042.4 ± 3817.4 ^b^
Mg	10,982.9 ± 1706.7 ^a^	10,136.2 ± 1530.4 ^a^	9043.5 ± 767.8 ^a^	9137.2 ± 1483.1 ^a^
Mn	1400.6 ± 351.2 ^ab^	1281.1 ± 394.6 ^a^	1313.0 ± 154.3 ^a^	1835.4 ± 231.9 ^b^
Na	1002.5 ± 80.5 ^a^	817.7 ± 66.0 ^a^	1054.5 ± 100.5 ^a^	958.7 ± 155.3 ^a^
P	18,455.5 ± 1182.6 ^a^	19,528.2 ± 2803.4 ^a^	20,712.3 ± 1131.2 ^a^	21,590.0 ± 3230.9 ^a^
S	3947.9 ± 79.2 ^a^	4254.9 ± 364.4 ^ab^	4429.7 ± 300.2 ^b^	4077.6 ± 187.1 ^ab^

**Table 2:** Target hazard quotient (THQ) for Zn content in the aboveground biomass of spinach. Data are presented as the mean ± standard deviation (SD) from four biological replicates. Different letters indicate statistically significant differences (*p* < 0.05) among the treatments of spinach aboveground biomass based on Fisher’s LSD test.

Treatments	THQ–Adults	THQ–Children
Control	0.024 ± 0.001^a^	0.065 ± 0.004^a^
Zn75	0.035 ± 0.004^b^	0.095 ± 0.011^b^
Zn150	0.055 ± 0.002^c^	0.147 ± 0.004^c^
Zn300	0.087 ± 0.009^d^	0.234 ± 0.024^d^

## Data Availability

The data presented in this study are available in this article.

## Supplementary Materials

The following supporting information can be downloaded at https://www.mdpi.com/article/10.3390/plants13233363/s1, Figure S1: Principal component analysis (PCA) of the water-soluble fraction of Zn and other nutrients in the substrate; Table S1: Content (mg kg^−1^ DW) of nutrients in the water-soluble fraction of the substrate; Table S2: Correlation of nutrients in the water-soluble fraction of the substrate; Table S3: Correlation of nutrients in the aboveground biomass of spinach.
