# Supplementary material for: The Dual Role of Zinc in Spinach Metabolism: Beneficial × Toxic
Source: Plants (Basel). 2024 Nov 29;13(23):3363. doi: 10.3390/plants13233363 (PMC11644758; doi:10.3390/plants13233363)
Supplement: Supplementary file 1 [file plants-13-03363-s001.zip › Table S1.pdf]

Table S1: Content (mg kg^{-1} DW) of nutrients in the water-soluble fraction of the substrate. Values represent the mean ± standard deviation (SD) of four biological replicates. Letters indicate significant differences ($p \leq 0.05$) between treatments as determined by Fisher's LSD test.

Nutrient (mg kg^{-1} DW)	Control	Zn75	Zn150	Zn300
Zn	0.26 ± 0.03^{a}	0.37 ±0.07^{b}	0.53 ± 0.09^{c}	0.98 ± 0.05^{d}
P	41.40 ± 0.96^{bc}	45.43 ± 2.80^{c}	37.06 ± 4.15^{ab}	35.87 ± 5.02^{a}
Mn	7.75 ± 0.51^{d}	5.55 ± 1.11^{c}	3.99 ± 0.58^{b}	2.77 ± 0.32^{a}
Ca	212.64 ± 9.65^{c}	145.59 ± 31.55^{b}	97.79 ± 8.33^{a}	83.67 ± 6.90^{a}
Cu	0.01 ± 0.001^{a}	0.01 ± 0.001^{a}	0.01 ± 0.002^{a}	0.01 ± 0.001^{a}
Fe	0.20 ± 0.02^{b}	0.18 ± 0.04^{ab}	0.16 ± 0.02^{a}	0.10 ± 0.004^{c}
K	92.38 ± 6.25^{c}	77.71 ± 15.41^{bc}	54.39 ± 9.73^{a}	67.64 ± 5.44^{ab}
Mg	110.75 ± 5.44^{c}	75.46 ± 22.16^{b}	64.93 ± 14.99^{ab}	45.34 ± 4.49^{a}
Na	8.94 ± 0.63^{b}	8.42 ± 1.45^{ab}	8.32 ± 1.83^{ab}	6.54 ± 0.90^{a}
S	16.91 ± 0.73^{a}	20.95 ± 6.10^{a}	20.02 ± 2.62^{a}	19.09 ± 5.90^{a}
