# Supplementary material for: The Dual Role of Zinc in Spinach Metabolism: Beneficial × Toxic
Source: Plants (Basel). 2024 Nov 29;13(23):3363. doi: 10.3390/plants13233363 (PMC11644758; doi:10.3390/plants13233363)
Supplement: Supplementary file 1 [file plants-13-03363-s001.zip › Table S2.pdf]

plants

MDPI

Table S2: Correlation of nutrients in the water-soluble fraction of the substrate. Values without asterisks are not statistically significant; * for $p < 0.05$; ** for $p < 0.01$; *** for $p < 0.001$.

	**Zn**	**P**	**Mn**	**Ca**	**Cu**	**Fe**	**K**	**Mg**	**Na**	**S**
Zn	x	−0.52*	−0.81***	−0.75**	0.41	−0.81***	−0.39	−0.73**	−0.52*	0.13
P	−0.52*	x	0.59*	0.50	−0.09	0.56*	0.49	0.43	0.51*	−0.02
Mn	−0.81***	0.59*	x	0.95***	−0.17	0.87***	0.77***	0.91***	0.58*	−0.13
Ca	−0.75**	0.50	0.95***	x	−0.26	0.80***	0.80***	0.96***	0.58*	−0.02
Cu	0.41	−0.09	−0.17	−0.26	x	−0.02	−0.05	−0.19	−0.14	0.47
Fe	−0.81***	0.56*	0.87***	0.80***	−0.02	x	0.59*	0.84***	0.63**	0.16
K	−0.39	0.49	0.77***	0.80***	−0.05	0.59*	x	0.71**	0.44	−0.05
Mg	−0.73**	0.43	0.91***	0.96***	−0.19	0.84***	0.71**	x	0.73**	0.06
Na	−0.52*	0.51*	0.58*	0.58*	−0.14	0.63**	0.44	0.73**	x	0.07
S	0.13	−0.02	−0.13	−0.02	0.47	0.16	−0.05	0.06	0.07	x
