# Supplementary material for: The Dual Role of Zinc in Spinach Metabolism: Beneficial × Toxic
Source: Plants (Basel). 2024 Nov 29;13(23):3363. doi: 10.3390/plants13233363 (PMC11644758; doi:10.3390/plants13233363)
Supplement: Supplementary file 1 [file plants-13-03363-s001.zip › Table S3.pdf]

plants

MDPI

Table S3: Correlation of nutrients in the aboveground biomass of spinach. Values without asterisks are not statistically significant; * for $p < 0.05$; ** for $p < 0.01$; *** for $p < 0.001$.

	**Zn**	**P**	**Mn**	**Ca**	**Cu**	**Fe**	**K**	**Mg**	**Na**	**S**
Zn	x	−0.57*	0.62*	−0.62**	−0.32	−0.92***	0.04	0.68**	−0.02	0.68**
P	−0.57*	x	0.22	−0.15	−0.54*	0.52*	0.77**	0.15	−0.24	−0.06
Mn	0.62*	0.22	x	−0.78***	−0.76**	−0.46	0.79***	0.80***	−0.56*	0.49
Ca	−0.62**	−0.15	−0.78***	x	0.90***	0.70**	−0.60*	−0.82***	0.10	−0.74**
Cu	−0.32	−0.54*	−0.76**	0.90***	x	0.42	−0.83***	−0.82***	0.12	−0.68**
Fe	−0.92***	0.52*	−0.46	0.70**	0.42	x	0.03	−0.72**	−0.31	−0.85***
K	0.04	0.77**	0.79***	−0.60*	−0.83***	0.03*	x	0.61*	−0.53*	0.27
Mg	0.68**	0.15	0.80***	−0.82***	−0.83***	−0.72**	0.61*	x	0.03	0.89***
Na	−0.02	−0.24	−0.56*	0.10	0.12	−0.31	−0.53*	0.03	x	0.45
S	0.68**	−0.06	0.49	−0.74**	−0.68**	−0.85***	0.27	0.89***	0.45	x
