# Supplementary figures and images for: The Dual Role of Zinc in Spinach Metabolism: Beneficial × Toxic
Source: Plants (Basel). 2024 Nov 29;13(23):3363. doi: 10.3390/plants13233363 (PMC11644758; doi:10.3390/plants13233363)

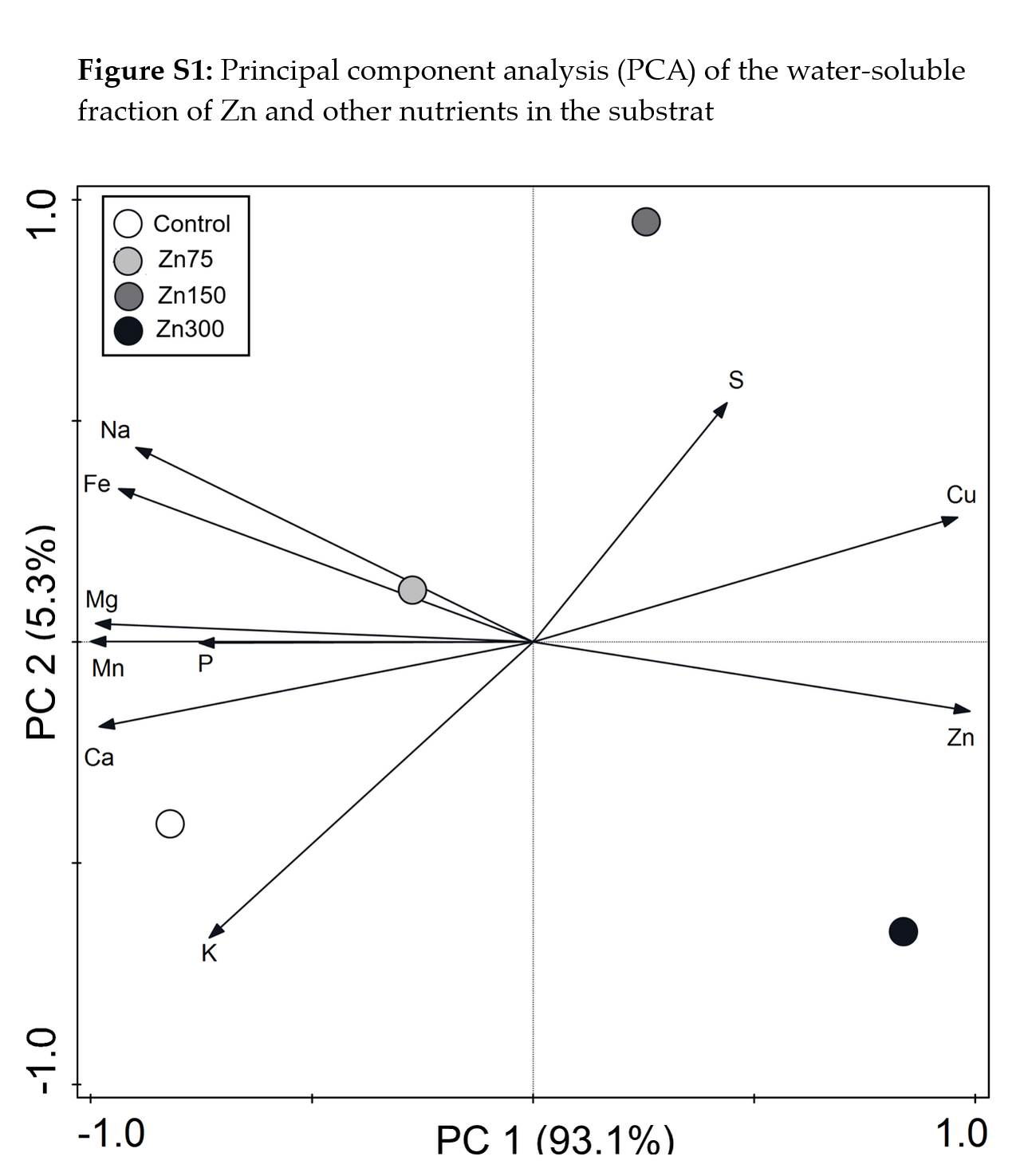

Supplement: Supplementary file 1 [file plants-13-03363-s001.zip › Figure S1.tif]
